# Cannabinoids and alcohol co-exposure modulate pathogen-induced pulmonary immune responses

**DOI:** 10.3389/fimmu.2025.1539813

**Published:** 2025-07-07

**Authors:** De’Jana Parker, Vijay Sivaraman

**Affiliations:** ^1^ Department of Molecular, Cellular and Developmental Biology, University of Michigan, Ann Arbor, MI, United States; ^2^ Department of Biological & Biomedical Sciences, North Carolina Central University, Durham, NC, United States

**Keywords:** cannabinoids, alcohol, pulmonary inflammation, polysubstance misuse, immune responses

## Abstract

Both alcohol and cannabinoid misuse cause substantial societal problems individually, and cannabis is the most popular illicit drug used simultaneously with alcohol. The role of endocannabinoids (eCB) and cognate receptors in the regulation of inflammation is clinically relevant; however, the role of cannabinoid receptors (CBRs) specifically in pulmonary inflammation and associated lung pathobiology remains elusive. For this study, we investigated the effects of binge cannabinoid exposure on pathogen-induced pulmonary inflammation. We also describe a binge ethanol + cannabinoid adolescent mouse model of pathogen-induced pulmonary inflammation by *Klebsiella pneumoniae* (*K. pneumoniae*) infection. We show that adolescent cannabinoid exposure primes the lung to a more severe inflammation in adulthood, and this response is mitigated by cannabinoid antagonists. We also show that ethanol and cannabinoid pre-exposure followed by microbial challenge yielded CBR-dependent pulmonary immune activation via danger-associated molecular pattern (DAMP) release. This research may shed light on CB signaling as it relates to DAMPs and can provide a framework to develop potential novel therapeutics in polysubstance use disorders.

## Graphical Abstract

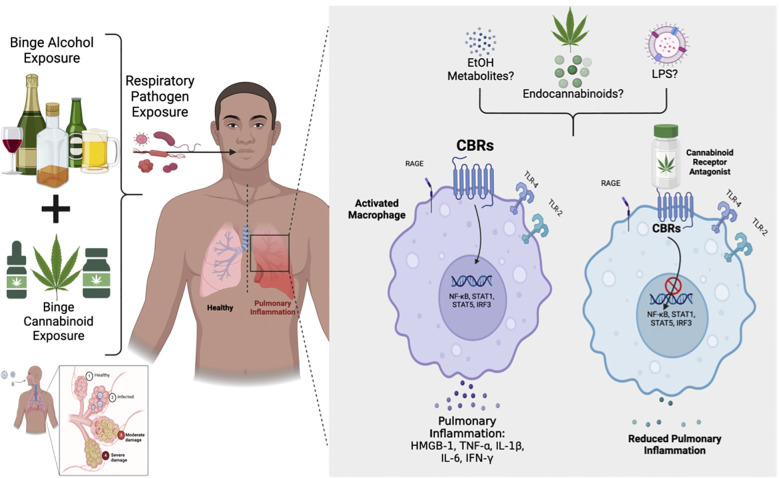

## Introduction

Cannabinoids (CBs) are the most widely used illicit substances in the world, with an estimated 188 million people reporting the use of cannabis in 2019. Its widespread use has raised many concerns over its long-term effects, particularly in the adolescent population (1). CBs are one of the most commonly used drugs among adolescents, with initial use beginning between the ages of 12 and 14 (2). *Cannabis sativa* is commonly inhaled by combustion of plant material or concentrates, but both are often inhaled via high-temperature vaporization using vaporizers and e-cigarettes or ingested as an extract, often in cannabis-infused foods (3–5). In addition to the plant *Cannabis sativa*, there are two classes of cannabinoids: the synthetic cannabinoids (e.g., WIN55212–2) and the endogenous cannabinoids (eCB), anandamide (ANA) and 2-arachidonoylglycerol (2-AG) (6). The eCB, CBRs, and enzymes/proteins responsible for their biosynthesis and degradation constitute the endocannabinoid system (ECS) (6–8).

The biological effects of cannabinoids are mainly mediated by two members of the G-protein-coupled receptor family, cannabinoid receptors 1 (CB1R) and 2 (CB2R). CB1R is abundantly expressed in the central nervous system, particularly in the neocortex, hippocampus, basal ganglia, and cerebellum. However, it is also present in the lungs, liver, and kidney. CB2R is often considered the peripheral CBR and is recognized for its expression on immune cells; however, it has also been identified on fibroblasts, chondrocytes, and neurons. In the lungs, CBR receptors can be found on structural cells and most leukocytes (8–10). A study of the distribution of CBRs found both CB1R and CB2R mRNA in the lungs and in bronchial tissue, with the CB1R mRNA levels being significantly higher than those of CB2R (6, 10–12). In addition, preliminary data from our lab show that CB1R is highly expressed, as well as CB2R, in mouse macrophages (13). CB1R and CB2R have been widely demonstrated to be important modulators of the immune system, potentially inducing immunosuppression (14).

Pulmonary inflammation is characterized by the activation of the innate immune system, as defined by increased numbers of innate immune cells in lung tissue (15). The first line of defense in the lungs against pathogens are epithelial cells and AMs. The airway epithelium forms the first barrier toward inhaled insults, separating the lung tissue from the environment. Consequently, epithelial cells are one of the first cells to be exposed to inhaled noxious gases and particles (16). In addition, increased numbers of macrophages are present in the airways, BALF, and sputum of patients with significant pulmonary inflammation. The enhanced numbers of macrophages are associated with respiratory disease severity (17).

Most of the pulmonary exacerbations that result in hospitalization are coupled with respiratory infections. Particularly in patients with severe respiratory complications, up to 78% of exacerbations are due to bacterial infections and viral infections (18). CB use has been shown to impair the lung’s defense against infection, implying that it might increase the risk of pneumonia (19, 20). In addition, CBs are frequently contaminated with potentially pathogenic bacteria—pathogens that could be inhaled into the lung and predispose someone to pneumonia (21, 22). *Klebsiella pneumoniae* is associated with increased bronchial and systemic inflammation and the development of pulmonary exacerbations (18, 23, 24). This can accelerate the progression of pulmonary inflammation through an increase in the frequency of exacerbations and through direct injury to the lung tissue (18, 25). Despite this data, very little research examining the effects of CB-induced inflammation and its role in respiratory infection has been done.

Both alcohol and cannabis misuse are significant societal problems individually, and cannabis is the most popular illicit drug used simultaneously with alcohol. Daily CB use has been shown to impair the lung’s defense against infection (19). Danger-associated molecular patterns (DAMPs) are proteins released from cells following an infection and are crucial inflammatory mediators. These proteins trigger the innate immune system upon release and induce a range of cellular responses to help clear out infections (15, 25–27). A role for DAMPs in the pathogenesis of pulmonary diseases such as ARDS has recently been proposed (28–30). Many studies have shown that the use of CBs increases susceptibility to respiratory infections. However, the exact mechanism through which exogenous and endogenous CBs modulate immune function is a subject of continued investigation. In addition, the immunomodulatory effects of alcohol and cannabinoids are not fully elucidated. Although polysubstance misuse of alcohol and cannabinoids is a huge societal problem, the role of both substances during infections has also not been fully studied. WIN55212–2 is a synthetic cannabinoid receptor agonist that selectively activates CB1 and CB2 receptors and is commonly used in experimental models to study the effects of cannabinoid receptor signaling independent of plant-derived or endogenous cannabinoids. We hypothesize that (1) adolescent intermittent synthetic cannabinoid receptor agonist (WIN55212-2) (AIC) exposure modulates the expression of proinflammatory cytokines which contributes to enhanced pulmonary inflammation and (2) EtOH-induced activation of eCB-CB1R and/or eCB-CB2R signaling is critical in the regulation of this cytokine balance in lung tissue.

## Materials and methods

### Synthetic cannabinoid receptor agonists and antagonist compounds used in the study

#### CBR1/2 dual agonist

WIN (WIN55212; (*R*)-(+)-[2,3-dihydro-5-methyl-3-(4-morpholinyl methyl)pyrrolo[1,2,3]-1,4-benzoxazin-6-yl]-1-naphthalenylmethanone mesylate) was purchased from Cayman Chemical (cat. #10736), and stock solutions were prepared following the manufacturer’s protocols. Briefly, WIN55212 (mesylate) is supplied as a crystalline solid. A stock solution was made by dissolving WIN55212 in DMSO (30 mg/mL) and then diluting with PBS in a 1:2 solution of DMSO/PBS (pH 7.2). WIN55212 is a synthetic agonist of cannabinoid receptors. Its mechanism of action involves mimicking endocannabinoids by activating CBR1 and CBR2, leading to the downstream modulation of neurotransmitter release, immune responses, inflammation, and cell signaling pathways.

#### CBR1 antagonist

CB1R, AM6545 (5-(4-[4-cyanobut-1-ynyl] phenyl)-1-(2,4-chlorophenyl)-4-methyl-N-(1,1-dioxo-thiomorpholino)-1H-pyrazole-3carboxamide) was purchased from Cayman Chemical (cat. #16316), and stock solutions were prepared following the manufacturer’s protocols. Briefly, AM6545 is supplied as a crystalline solid. A stock solution was prepared by dissolving the AM6545 in DMSO (10 mg/mL) and then diluting it with PBS in a 1:2 solution (pH 7.2).

#### CBR2 antagonist

SR144528 (N-[(1S)-endo-1,3,3-trimethylbicycloheptan2-yl]-5-(4-chloro-3-methylphenyl)-1-[(4-methyl phenyl)methyl]-1H-pyrazole-3-carboxamide) was purchased from Cayman Chemical (cat. #9000491), and stock solutions were prepared following the manufacturer’s protocols. Briefly, SR 144528 is supplied as a crystalline solid. A stock solution was prepared by dissolving SR 144528 in ethanol (30 mg/mL) and then diluting in PBS in a 1:1 solution of ethanol/PBS (pH 7.2).

##### Animal treatment

Mice (C57-BL/6J) were purchased from Jackson Laboratories (Bar Harbor, MA, USA). Adolescent (6-week-old male and female) mice were used for all of the experiments. Mice at 6 weeks of age exhibit physiological and neurological development that align with the typical human adolescent period, spanning approximately 12 to 18 years. Consequently, they serve as a pertinent model to investigate the impact of alcohol and cannabinoids on a developing system. At 6 weeks, the mice have a functional but still maturing immune system, making them an ideal model to investigate how alcohol and cannabinoids modulate pulmonary immune responses. This is particularly relevant for understanding lung inflammation. Lastly, using 6-week-old mice ensures that experiments are conducted before they reach full adulthood, thereby minimizing the variability caused by age-related immune changes. This approach also aligns with ethical guidelines to minimize distress and maintaining consistent experimental conditions.

After delivery, the mice were allowed to recover from shipping stress for 1 week at the NC Central Univ. Animal Resource Complex, which is accredited by the American Association for Accreditation of Laboratory Animal Care. All animal care and use were conducted in accordance with the Guide for the Care and Use of the Laboratory Animals (National Institutes of Health) and approved by the NCCU IACUC. The mice were maintained at 25°C and 15% relative humidity with alternating 12-h light/dark periods. The intermittent binge-drinking model used in this study was developed by White and Swartzwelder (31). Briefly, 100 proof EtOH was diluted in autoclaved (sterile) water to a concentration of 20% (v/v) and was administered by oral gavage using an 18-gauge stainless steel gavage needle. Each mouse received a dose of 5 g/kg of alcohol or PBS (mock control). At time points of EtOH exposure, a cohort of mice (both EtOH and vehicle-treated) was exposed to CBR dual agonist, CB1R, or CB2R antagonists (1 mg/kg, i.p.) 30 mins before each EtOH dose. We selected this dose based on its utilization in previous research studies (32). All control and treatment groups comprised *N* = 10 mice per group unless otherwise noted. C57BL/6 mice were administered either the cannabinoid agonist WIN or PBS (IP) on an alternating day schedule for 10 days. At each dosing time point, a subset of animals (*n* = 5) also received CB1R or CB2R antagonist (1 mg/kg, IP), administered 20 min prior to each WIN exposure. After a 12-day rest period following the final antagonist and agonist exposure, all mice were challenged intranasally with a sub-lethal dose of *Klebsiella pneumoniae*. After 1 h and 24 h post-alcohol exposure, tail blood was obtained from mice to confirm the blood alcohol concentration (BAC). At 1-h post-alcohol exposure, the BAC ranged 100–250 mg/dL (0.1%–0.25%), and by 24 h post-treatment, the BAC was below detection level. Though the range of BAC within mice was ~2.5 times higher than the human legal level of intoxication (0.08), it can be noted that the metabolism of EtOH in mice is reported to occur more rapidly, and this animal alcohol exposure is a well-accepted model for binge alcohol exposure studies (33). At time points of experimental completion, the mice were humanely sacrificed using CO_2_ asphyxiation, as per the accepted animal protocol (34).

##### Bacterial challenge

*Klebsiella pneumoniae* (*K. pneumoniae*) was used for the pulmonary challenge through nasal drip. *K. pneumoniae* was streaked on brain heart infusion broth (BHI) agar plates and grown overnight in an incubator at 37°C. On the next day, one isolated colony from the plate was transferred into 20 mL and grown overnight in BHI broth with constant shaking at 37°C. The mice were sedated with an i.p. injection of ketamine (100 mg/kg) and xylazine (50 mg/kg) and infected IN with a sub-lethal dose (2 × 10^4^ CFU) of *K*. *pneumoniae* suspended in 20 μL 1× phosphate-buffered saline (PBS) (35, 36). Body weight and health conditions were monitored daily per IACUC protocols. Humane endpoints were not used for this study as experimental time points of completion were chosen before any significant body weight change or clinical signs of disease could be observed. At time points of experimental completion (24 hpi), the mice were humanely euthanized using CO_2_ asphyxiation and cervical dislocation, as per our accepted animal protocol and Animal Resource Complex housing guidelines and conditions.

Bacterial burden from lungs and spleen: *Klebsiella pneumoniae* (cat. #BA-1705) was purchased from the American Type Culture Collection (ATCC, Manassas, VA, USA). The bacterial concentration for *Klebsiella pneumoniae* (*K. pneumoniae*) was determined by measuring the optical density (OD) at 600 nm and diluting to a working solution (colony-forming units/mL). An initial series of pilot studies was conducted to determine the optimal dose of *K. pneumoniae* via intranasal inoculation to achieve an appropriate lung infection model using both male and female mice. Inoculation was performed on day 0, followed by tissue collection at 24 and 48 h to evaluate changes in body weight, sickness symptoms, survival, and lung bacterial load while testing several doses of *K. pneumoniae* (37). Briefly, *K. pneumoniae* was used for the pulmonary challenge through nasal drip. *K. pneumoniae* was streaked on brain heart infusion broth (BHI) agar plates and grown overnight in an incubator at 37°C. On the next day, one isolated colony from the plate was transferred into 20 mL and grown overnight in BHI broth with constant shaking at 37°C. The optimum dose of 2 × 10^9^ CFU/mL was calculated for each mouse. The concentration of the bacteria was determined by using a Thermoscientific BioMate 35 spectrophotometer at 600 OD. The mice were sedated with an i.p. injection of ketamine (100 mg/kg) and xylazine (50 mg/kg) and infected intra-nasal (IN) administration with a sub-lethal dose of *K. pneumoniae* suspended in 20 μL 1× phosphate-buffered saline (PBS). *K. pneumoniae* was washed in PBS before infection (35, 36). Body weight and health conditions were monitored daily per IACUC protocols. Humane endpoints were not used for this study as experimental time points of completion were chosen before any significant body weight change or clinical signs of disease could be observed. At time points of experimental completion (24 hpi), the mice were humanely euthanized using CO_2_ asphyxiation and cervical dislocation, as per our accepted NCCU animal protocol and NCCU Animal Resource Complex housing guidelines and conditions.

##### Wet/dry lung ratio

The lungs were immediately removed from euthanized mice (24 h post-infection) and weighed (wet weight). The lung tissue was then dried in an incubator (65°C) for 24 h and reweighed (dry weight). The wet/dry ratio was calculated by dividing the wet weight by the dry weight with an increase in this ratio being a preclinical metric of acute lung injury and reflecting severity of lung inflammation.

Sample preparation from lungs: Bronchoalveolar lavage fluid (BALF) fluid was obtained per the established protocol (38). In brief, the mice were euthanized, and a catheter attached to a 1-mL syringe was inserted into the trachea. The syringe was then used to deliver 1× PBS, which was gently pipetted up and down 3× to remove the fluid. BALF was then directly utilized for flow studies or clarified via centrifugation for cytokine analysis. Alternatively, the total lung was dissected out, then homogenized in PBS containing protease inhibitors, clarified via centrifugation, and quantified using the BCA assay.

##### Histopathology

After experimental completion, the mice were humanely euthanized using a CO_2_ chamber. Lung pathology was evaluated via histopathology as previously described. Briefly, after dissecting and exposing the trachea, a catheter connected to a syringe was inserted into the trachea to inflate the lungs with 1 mL of 10% formalin. The inflated lungs were collected and suspended in 10% formalin for 12 h. The lungs were washed in 1X PBS, placed in histology cassettes, and immersed in 70% EtOH. Tissues were embedded in paraffin, sliced (three 5-μm sections 200 μm apart per lung), and stained using hematoxylin/eosin. Histology was scored for lung damage using a rubric created by the American Thoracic Society as previously described (39).

##### Luminex analysis

Cytokine protein levels were assessed by performing Luminex analysis upon lung lysate, using Millipore reagents and a Luminex 200 system (Millipore Sigma, Burlington, MA, USA). Briefly, Millipore MILLIPLEX^®^ MAP mouse cytokine/chemokine magnetic bead panel 96-well plate assay catalog number MCYTOMAG-70K was utilized for cytokine analysis. Whole lungs were homogenized in 1 mL of PBS. The samples were then centrifuged to remove debris. The supernatant was diluted using a 1:5 dilution in DMEM media. Luminex runs according to the standard protocol. To prepare pre-mixed antibody-immobilized beads, the beads were sonicated for 30 s and then vortexed for 1 min. First, 200 µL of wash buffer was added to each well and placed on a place shaker for 10 min at RT. Next, the wash buffer was removed, and 25 µL of the standard was added to appropriate wells, an assay buffer was used for the 0 pg/mL standard, and 25 µL of the assay buffer was added to the sample wells. Then, 25 µL of matrix solution was added to the background, standard, and control wells. Next, 25 µL of the sample was added to appropriate wells, and 25 µL of beads was added to each well. The plate was then sealed, wrapped in foil, and incubated with agitation overnight at 2°C –8°C. On the following day, the well contents were removed, and the plate was washed two times. Furthermore, 25 µL of RT detection antibodies was then added to each well. The plate was then sealed, covered in foil, and incubated with agitation for 1 h RT. Next, 25 µL of streptavidin–phycoerythrin was added to each well, and the plate was sealed, covered in foil, and incubated for 30 min RT. Afterward, the well contents were removed and the plate was washed two times. Then, 150 µL of sheath fluid was added to each well, and the beads were resuspended on the plate shaker for 5 min. Lastly, the plate was run on Luminex 200™.

##### ELISA analysis

The whole lung was homogenized in 1 mL of PBS containing protease inhibitor. Following BD OptEIA™ Mouse ELISA Set, the homogenized lung was analyzed. First, 100 µL of 1:250 diluted capture antibody was added to each well and incubated overnight at 4°C. On the next day, the wells were aspirated and washed with wash buffer three to five times. Then, the plates were blocked using 200 µL of Assay Diluent (PBS with 10% FBS) and incubated for 1 h at RT. The following blocking is another aspirate/wash step. Next, 100 µL of standard or sample was added to each well and incubated for 2 h at RT. The plate was then aspirated and washed five times. A 100-µL working detector was then added to each well and incubated for 1 h at RT. The plate was then aspirated and washed seven to 10 times. Then, 100 µL of substrate solution (BD OptEIA TMB Substrate Reagent Set) was added to each well and incubated for 30 min at RT in the dark. Lastly, 50 µL of stop solution of twofold dilution of 1 M HCL and water was added to each well, and the absorbance was read at 450 nm within 30 min.

##### Flow cytometry

Cells isolated from BAL were stained with fluorescent-tagged antibodies as per the manufacturers’ instructions. CD170 (SiglecF)-FITC, Ly6G-PE, F4/80-AF700, CD11B-APC, CD11C-PC5.5A, and eBioscience Fixable Viability Dye eFluor 450 were purchased from Thermo Fisher Sci (eBiosciences). In brief, antibodies were chosen to sort mouse dendritic cells, exudative macrophages, alveolar macrophages, and neutrophils. Data acquisition was performed using a three-color CytoFLEX flow cytometer (Beckman Coulter, FL, USA). The CytExpert acquisition software (Beckman Coulter) was used to acquire samples, while FCS Express (version 7; DeNovo Software, Pasadena, CA, USA) was then used for the analysis. For each sample, at least 30,000 events were collected.

Statistical analysis: All data are presented as mean ± standard error of the mean (SEM). For comparisons between two groups, statistical significance was determined using an unpaired Student’s *t*-test. For experiments involving more than two groups, one-way ANOVA followed by Tukey–Kramer *post hoc* testing was used to assess pairwise differences. When analyzing experiments with two independent variables and potential interaction effects, a two-way ANOVA was performed, followed by Bonferroni *post hoc* testing. If the data did not meet the assumptions of normality, a non-parametric analysis was conducted using the Kruskal–Wallis test. Statistical analyses were performed using GraphPad Prism version 10 (GraphPad Software, San Diego, CA, USA). A *p*-value <0.05 was considered statistically significant.

## Results

**Figure 1:**
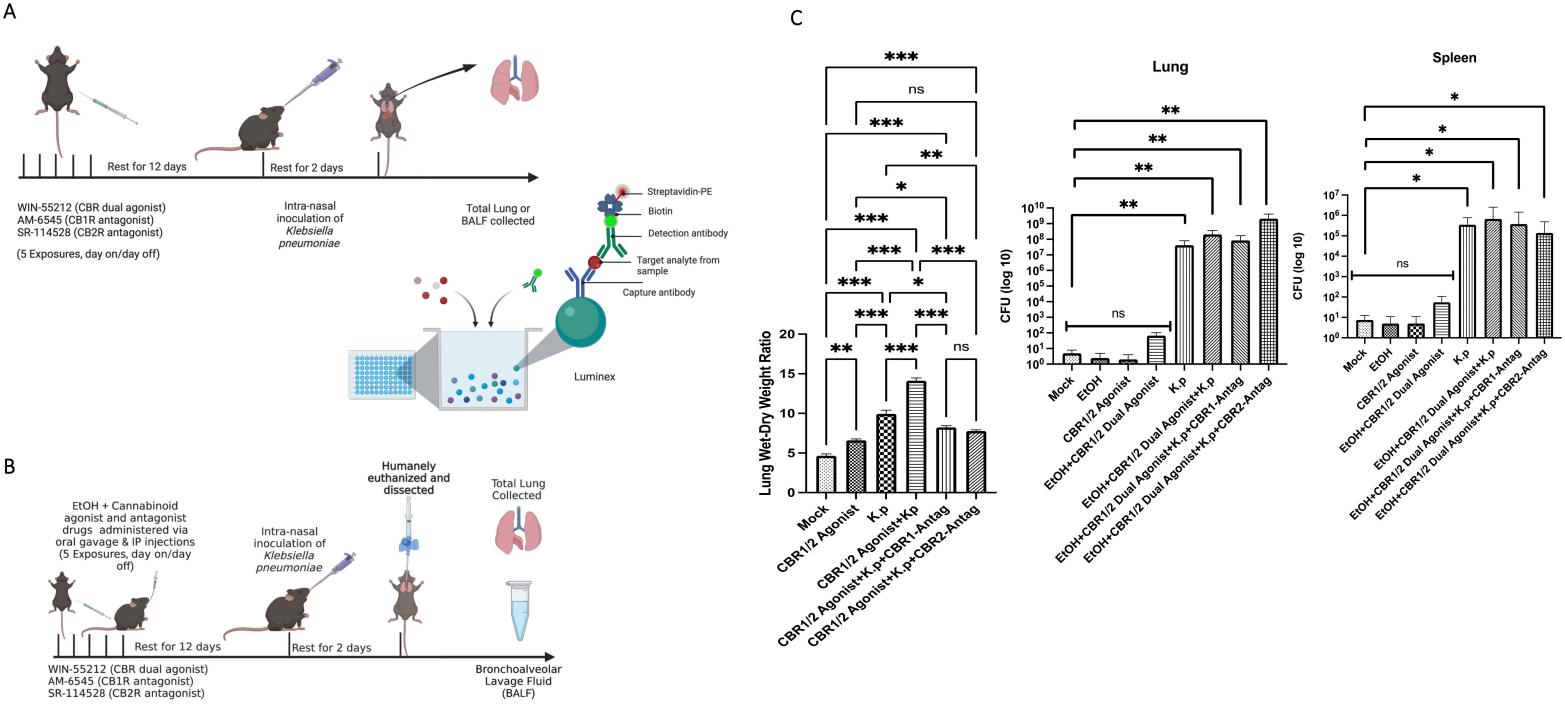
Adolescent intermittent EtOH + synthetic cannabinoid receptor agonist (WIN55212-2) mouse model. **(A)** C57-BL6 mice were exposed either to cannabinoid agonist WIN or PBS (IP) day on/day off for 10 days. At each time point of dosing, a subset of animals were exposed to CB1R or CB2R antagonist (1 mg/kg body weight) (IP) 20 min before alcohol exposure. After 12 days of rest post-last binge and antagonist exposure, the mice were challenged with a sublethal intranasal dose of *K*. *pneumoniae.* The mice were humanely euthanized at 48 h, and total lungs were collected and assessed via Luminex assay. **(B)** Study design: C57-BL6 mice were exposed either to EtOH and/or synthetic cannabinoid receptor agonist (WIN55212-2) or PBS (IP) day on/day off for 10 days. At each time point of dosing, a subset of animals was exposed to CBR1 or CBR2 antagonist (1 mg/kg body weight) (IP) 20 min before alcohol exposure. After 12 days of rest post-last binge and antagonist exposure, the mice were challenged with a sublethal intranasal dose of *K*. *pneumoniae.* The mice were humanely euthanized; the bronchoalveolar lavage fluid (BALF) and total lungs were collected and assessed. **(C)** Lung wet/dry ratio of mice pre-exposed to synthetic cannabinoid receptor agonist (WIN55212-2), cannabinoid antagonist, and then infected with a sub-lethal dose of *Klebsiella pneumoniae*. *n* = 3 mice per treatment group. Bacterial burden calculation from mice treated with EtOH and synthetic cannabinoid receptor agonist (WIN55212-2) challenged with *K*. *pneumoniae*. *=0.05, **=0.001 and ***=0.0001. ns, not significant.

First, we evaluated the inflammatory cytokine/chemokine profile from adolescent mice treated with synthetic cannabinoid receptor agonist (WIN55212-2) using a microbial challenge mouse model. C57-BL6 mice were given five doses of synthetic cannabinoid receptor agonist (WIN55212-2) or cannabinoid antagonist CB1R (AM-6545) or CB2R (SR-114528) via i.p. injection day on/day off over 12 days. At 12 days post-last binge and antagonist exposure, the mice were challenged with a sublethal intranasal dose of *K. pneumoniae*. The mice were humanely euthanized at 48 hpi, and the total lungs were collected and assessed via Luminex (**Figure 1A**). Next, we wanted to evaluate the inflammatory cytokine/chemokine profile from adolescent mice treated with both EtOH and synthetic cannabinoid receptor agonist (WIN55212-2)s (polysubstance misuse) using a microbial challenge mouse model. The C57-BL6 mice were given five doses of binge EtOH (oral gavage), cannabinoid dual agonist WIN-55212, or cannabinoid antagonist CB1R (AM-6545) or CB2R (SR-114528) via i.p. injection day on/day off over 12 days. At 12 days post-last binge and antagonist exposure, the mice were challenged with a sublethal intranasal dose of *K. pneumoniae*. The mice were humanely euthanized at 48 hpi, and the total lungs were collected and assessed via ELISA (**Figure 1B**).

### Synthetic cannabinoid receptor agonist (WIN55212-2) pre-exposure exacerbates *K. pneumoniae*-induced lung pathology

We evaluated next the wet/dry lung ratio from the lungs of synthetic cannabinoid receptor agonist (WIN55212-2)-exposed mice. An increase in wet/dry lung ratio is a well-described preclinical metric of acute lung injury and reflects the severity of lung inflammation. After dissection, the lungs were weighed both wet and after drying, and the data was used to calculate the ratio (35, 40). From these observations, it could be noted that the lungs from the synthetic cannabinoid receptor agonist (WIN55212-2) agonist treatment groups followed by bacterial infection mice had significantly higher wet/dry ratios compared to the control groups (bacterial infection alone). Most interestingly, the groups treated with synthetic cannabinoid receptor agonist (WIN55212-2) receptor antagonists had lower wet/dry ratios compared to synthetic cannabinoid receptor agonist (WIN55212-2) agonist infection (*p* < 0.001). Bacterial burden was determined by homogenizing the whole lungs and spleen and plating them using 10-fold serial dilutions. After 48 h of incubation time, the synthetic cannabinoid receptor agonist (WIN55212-2)-exposed mice inoculated with *K. pneumoniae* had a statistically significant increase in the number of bacterial colonies compared to the control samples for both the lung and spleen (**Figure 1C**).

**Figure 2:**
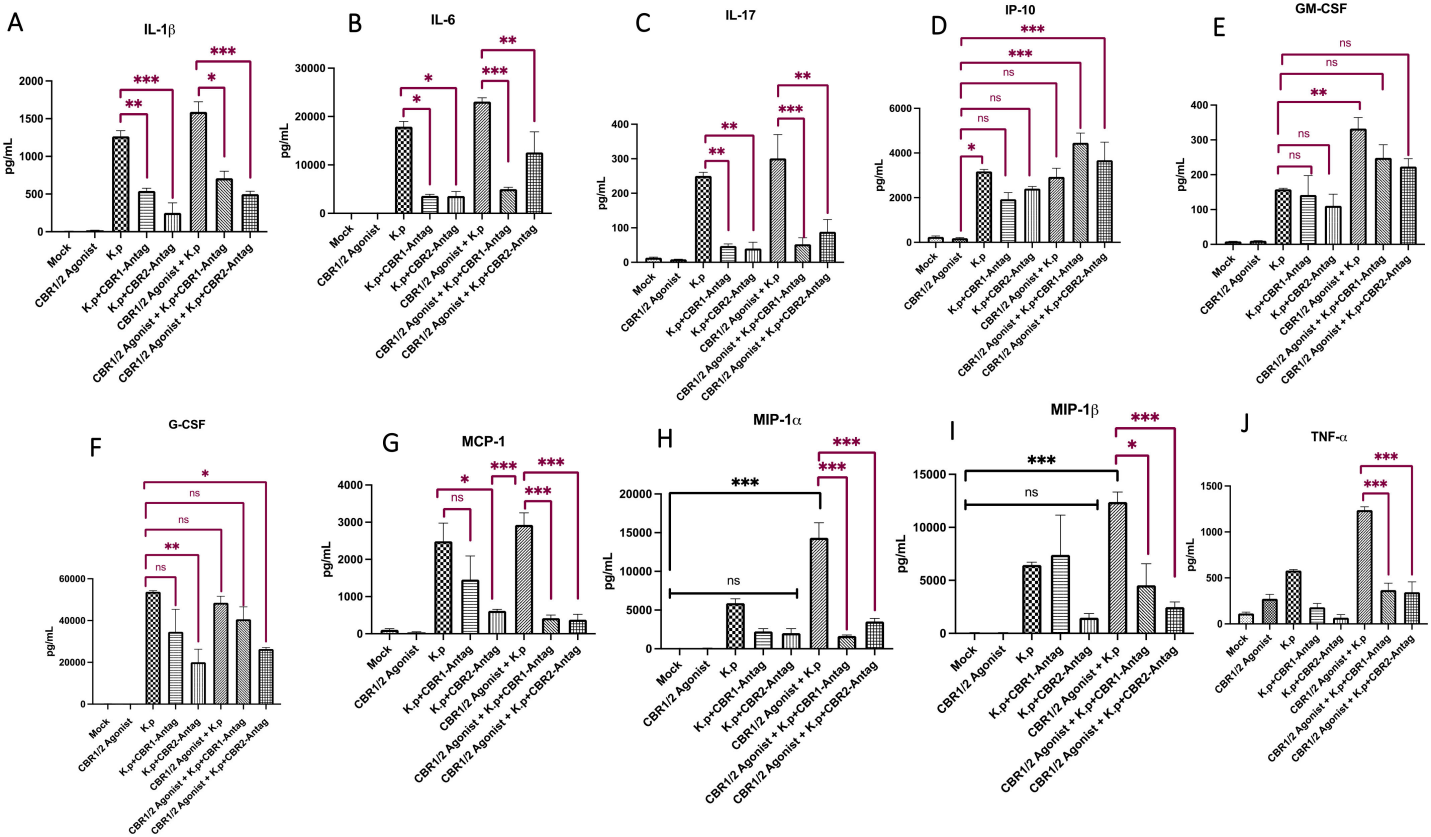
Lung cytokine and chemokine profiling following synthetic cannabinoid receptor agonist exposure and *Klebsiella pneumoniae* infection. Whole-lung homogenates were collected 48 h post-infection and analyzed for cytokine and chemokine expression using Luminex multiplex analysis. Pro-inflammatory cytokines IL-1β, IL-6, and IL-17 **(A–C)**, chemokine IP-10 **(D)**, and colony-stimulating factors G-CSF and GM-CSF **(E, F)** were quantified, along with MCP-1, MIP-1α, MIP-1β, and TNF-α **(G–J)**. Data represent protein concentrations in total lung tissue and reflect inflammatory responses across treatment groups. The mice were pre-treated with cannabinoid receptor agonist WIN or PBS (IP) for 10 days (day on/day off), with or without co-administration of CB1R or CB2R antagonists 20 min prior to WIN. After a 12-day rest period, the mice were intranasally infected with *K. pneumoniae* and euthanized 48 h later for tissue collection. Each treatment group consisted of *n* = 3 mice. Data are presented as mean ± SEM. Statistical comparisons were performed using one-way ANOVA followed by Tukey–Kramer *post hoc* test to assess differences among treatment groups. Comparisons to the mock-infected group are indicated with black significance bars, and comparisons to the *K. pneumoniae*-infected control group are indicated with red significance bars. A *p*-value <0.05 was considered statistically significant. *=0.05, **=0.001 and ***=0.0001. ns, not significant.

Next, the total lung from mice was evaluated for the presence of inflammatory cytokines by Luminex analysis. We evaluated the protein expression of the following interleukins: IL-1β, IL-6, and IL-17 (**Figures 2A–C**). As expected, the innate immune cytokines IL-1β and IL-6 were upregulated in the *K*. *pneumoniae*-infected lungs pretreated with synthetic cannabinoid receptor (WIN55212-2) agonist compared to mock controls and *K. pneumoniae* control. Most interestingly, the lungs from animals treated with *K. pneumoniae*, synthetic cannabinoid receptor agonist (WIN55212-2) agonist, and cannabinoid receptor antagonists showed a diminishment in protein expression for IL-1β, IL-6, and IL-17. For IL-1β, the highest amount (in pg/mL) was present in the CB-agonist + *K. pneumoniae* group. Interestingly, the levels of IL-1β decrease in groups treated with CB1/2 antagonists (CB-agonist + *K. pneumoniae* vs. CB-agonist + *K. pneumoniae* + CB1R-antag, *p* < 0.05; CB-agonist + *K. pneumoniae* vs. CB-agonist + *K. pneumoniae* + CB2R-antag, *p* < 0.001). The same trend is present for IL-6 with the highest amount (in pg/mL) present in the CB-agonist + *K. pneumoniae* group and the levels decreasing in groups treated with CB1/2 antagonists (CB-agonist + *K. pneumoniae* vs. CB-agonist + *K. pneumoniae* + CB1R-antag, *p* < 0.001; CB-agonist + *K. pneumoniae* vs. CB-agonist + *K. pneumoniae* + CB2R-antag, *p* < 0.01). Surprisingly, the IL-17 levels were also observably activated in the *K*. *pneumoniae*-infected animals pretreated with synthetic cannabinoid receptor agonist (WIN55212-2) agonist (CB-agonist + *K. pneumoniae* vs. CB-agonist + *K. pneumoniae* + CB1R-antag, *p* < 0.001; CB-agonist + *K. pneumoniae* vs. CB-agonist + *K. pneumoniae* + CB2R-antag, *p* < 0.01), suggesting a potential role for other inflammatory cells such as natural killer T cells. IL-2 and IL-15 did not have statistical significance (data not shown).

We next evaluated the protein expression levels of interferon gamma-induced protein (IP-10) (**Figure 2D**). IP-10 is a small protein secreted by many cells in response to IFN-γ. Interestingly, the levels of IP-10 (in pg/mL) were lowest in the animals treated with CB-agonist compared to the *K. pneumoniae* control. More interestingly, there was a statistically significant increase in IP-10 in mice treated with CB- agonist + *K. pneumoniae* +CB1R-tag compared to the CB-agonist control (*p* < 0.001) and CB- agonist + *K. pneumoniae* + CB2R-tag compared to the CB-agonist control (*p* < 0.001).

We also evaluated the protein levels from the cytokines granulocyte colony-stimulating factor (G-CSF) and granulocyte–macrophage colony-stimulating factor (GM-CSF) (**Figures 2E, F**). For both G-CSF and GM-CSF, the levels were statistically different in the mock controls vs. treatment groups, suggesting activation in *K. pneumoniae*-infected animals pretreated with synthetic cannabinoid receptor agonist (WIN55212-2). Interestingly, the levels of G-CSF (in pg/mL) were lowest in animals treated with *K. pneumoniae* + CB2R-antagonists (*K. pneumoniae* vs. *K. pneumoniae* + CB2R-antag, *p* < 0.01). For GM-CSF, the levels were statistically different in the *K. pneumoniae* control vs. CB-agonist + *K. pneumoniae* (*p* < 0.01). Although the lungs from animals treated with *K. pneumoniae* + cannabinoid agonist + CB1/2R antagonists showed a diminishment in protein expression compared to *K. pneumoniae* + CB-agonist groups for both G-CSF and GM-CSF, this response was not statistically significant.

We also evaluated the protein levels from the following cytokines: monocyte chemoattractant protein (MCP-1), macrophage inflammatory protein-alpha (MIP-1α), and macrophage inflammatory protein-beta (MIP-1β), and tumor necrosis factor-alpha and (**Figures 2G–J**). Interestingly and fitting with our hypothesis, the levels of MCP-1, MIP-1α, and MIP-1β (in pg/mL) were greatest in mice treated with CB-agonist + *K. pneumoniae* but statistically lower in mice treated with CB-agonist + *K. pneumoniae* + CBR-antagonists (MCP-1: CB-agonist + *K. pneumoniae* vs. CB-agonist + *K. pneumoniae* + CB1R-antag, *p* < 0.001; MCP-1: CB-agonist + *K. pneumoniae* vs. CB-agonist + *K. pneumoniae* + CB2R-antag, *p* < 0.001; MIP-1α: CB-agonist + *K. pneumoniae* vs. CB-agonist + *K. pneumoniae* + CB1R-antag, *p* < 0.001; MIP-1α: CB-agonist + *K. pneumoniae* vs. CB-agonist + *K. pneumoniae* + CB2R-antag, *p* < 0.001; MIP-1β: CB-agonist + *K. pneumoniae* vs. CB-agonist + *K. pneumoniae* + CB1R-antag, *p* < 0.05; MIP-1β: CB-agonist + *K. pneumoniae* vs. CB-agonist + *K. pneumoniae* + CB2R-antag, *p* < 0.001). The levels of TNF-α follow the same trends (CB-agonist + *K. pneumoniae* vs. CB-agonist + *K. pneumoniae* + CB1R-antag, *p* < 0.001; CB-agonist + *K. pneumoniae* vs. CB-agonist + *K. pneumoniae* + CB2R-antag, *p* < 0.001).

### Synthetic cannabinoid receptor agonist (WIN55212-2) exposure exacerbates key clinical features of microbial pneumonia, and this response is mitigated by CBR antagonists

**Figure 3:**
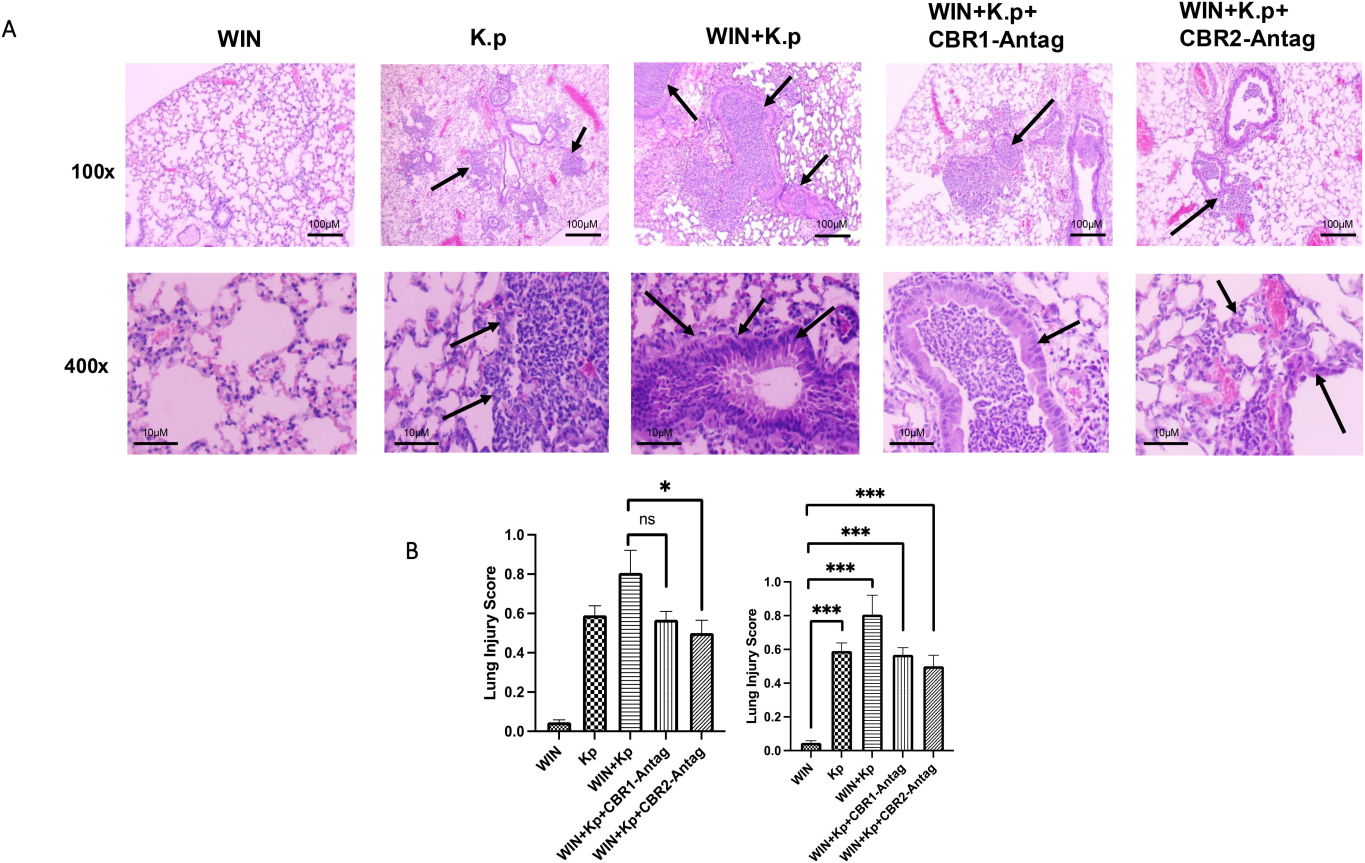
Synthetic cannabinoid receptor agonist (WIN55212-2) exacerbates lung pathology during *Klebsiella pneumoniae* infection, while CBR antagonists mitigate this response. **(A)** Representative H&E-stained lung sections (×100 and ×400 magnification) from ethanol-treated mice infected with *K*. *pneumoniae* and exposed to WIN55212-2, with or without cannabinoid receptor antagonists. Arrows indicate alveolar wall thickening and neutrophil infiltration. *n* = 4 mice per group. **(B)** Acute lung injury (ALI) scores were assessed using an ATS-established histopathological rubric to quantify inflammation and tissue damage across treatment groups. *=0.05 and ***=0.0001. ns, not significant.

The most evident evidence of a role for synthetic cannabinoid receptor agonist (WIN55212-2) receptors within pulmonary inflammation was observed from the histopathology of lungs using H&E staining of formalin-fixed sections. Prior data suggest an increased cytokine burden that could contribute to worsened disease post-infection. We observed neutrophil chemotaxis in the lungs of *K. pneumoniae-infected* animals. The foci of inflammation surrounded by multi-nucleated cells and alveoli thickening was observed. The inflammatory response was increased in CB agonist (WIN) + *K. pneumoniae*-exposed animals. However, the neutrophilic influx was significantly diminished in the lungs of animals treated with CBR antagonists (**Figure 3A**). The images were scored using the American Thoracic Society (ATS) scoring rubric to provide quantitation of inflammatory foci using acute lung injury parameters (**Figure 3B**).

### Evaluating the pulmonary effects of EtOH and synthetic cannabinoid receptor agonist (WIN55212-2) co-exposure (polysubstance misuse) using a microbial challenge model

DAMPs are proteins released from cells after infection. These proteins are critical inflammatory mediators. DAMPS activate innate immune cells upon release and can induce a wide range of cellular responses that help clear out infections such as the release of proinflammatory cytokines. High mobility group box 1 (HMGB-1) is a nonhistone chromatin-associated protein and representative DAMP. HMGB-1 normally exists as a nuclear protein but can be secreted into the extracellular environment through passive or active release. Extracellular HMGB-1 can bind to several different receptors and often binds to either receptor for advanced glycosylation end product (RAGE) receptor or pattern recognition receptors (PRRs) Toll-like receptor 2 and 4 found on the plasma membrane, which leads to the NFk-B-dependent release of proinflammatory cytokines. Interleukin-6 (IL-6), interleukin-1 beta (IL-1β), and tumor necrosis factor-alpha (TNF-α) are proinflammatory cytokines often released in response to immune activation (41, 42).

**Figure 4:**
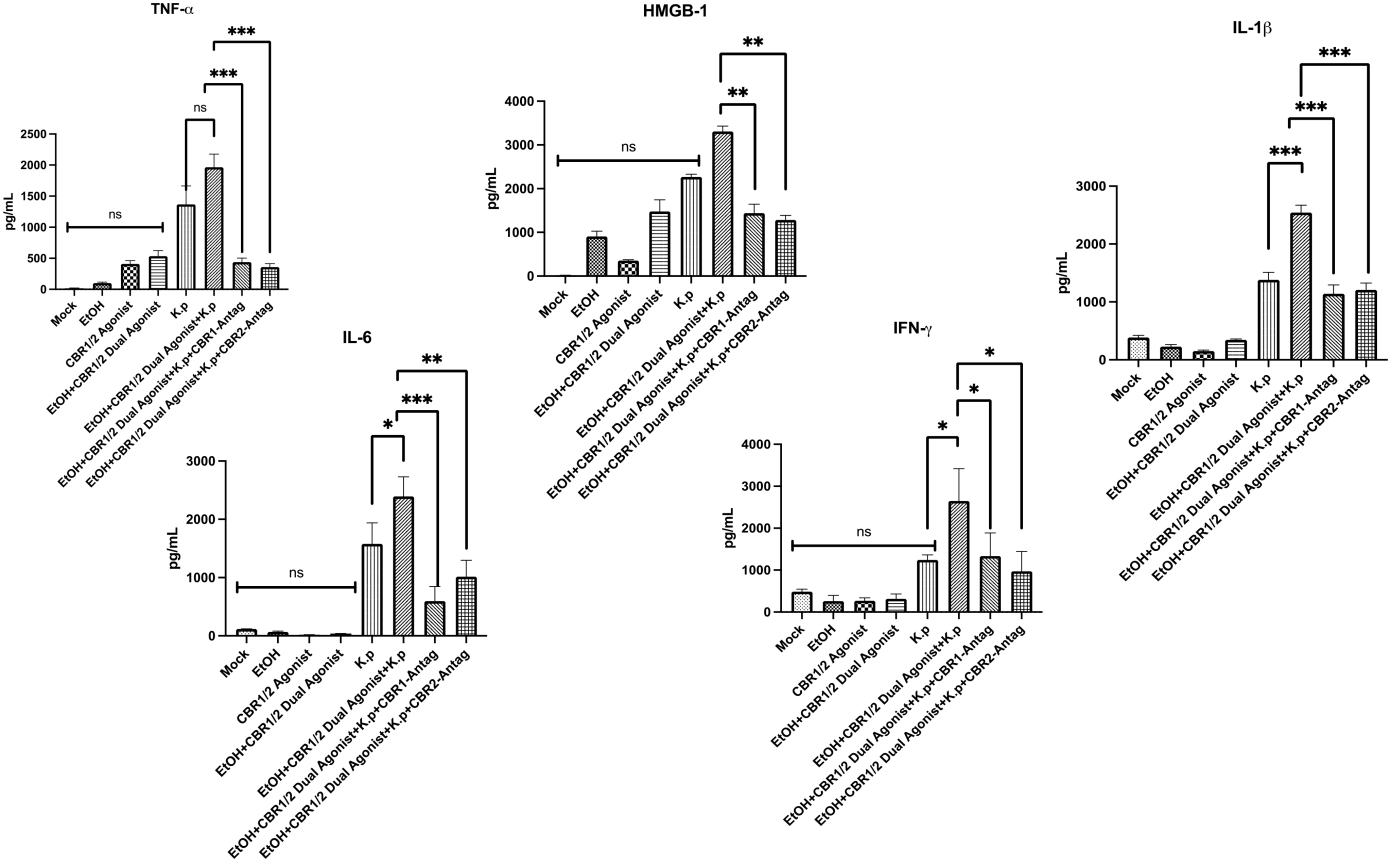
Evaluation of DAMP-associated inflammatory markers in BALF following alcohol and WIN55212–2 co-exposure during *Klebsiella pneumoniae* infection. Damage-associated molecular patterns (DAMPs) are endogenous danger signals released during infection and cellular stress that can amplify innate immune responses. To evaluate DAMP-associated signaling in pulmonary inflammation, bronchoalveolar lavage fluid (BALF) was collected from mice 48 h after *Klebsiella pneumoniae* infection. The mice were pre-treated with the synthetic cannabinoid receptor agonist WIN55212-2 (WIN) or PBS via intraperitoneal injection on an alternating day schedule for 10 days. In select groups, either a CB1R or CB2R antagonist (1 mg/kg, IP) was administered 20 min prior to each WIN dose. Following a 12-day rest period after the final exposure, all mice were intranasally challenged with a sublethal dose of *K. pneumoniae*. The protein levels of high mobility group box 1 (HMGB-1), a representative DAMP, and proinflammatory cytokines IL-6, IL-1β, and TNF-α were quantified in BALF using enzyme-linked immunosorbent assay (ELISA). These measurements were used to assess the extent of inflammatory signaling in response to cannabinoid and alcohol co-exposure. Each treatment group included *n* = 3 mice. Data are presented as mean ± SEM. Statistical comparisons between groups were performed using one-way ANOVA followed by Tukey–Kramer *post hoc* testing to assess significant differences. A *p*-value <0.05 was considered statistically significant. *=0.05, **=0.001 and ***=0.0001. ns, not significant.

HMGB-1 is also an important DAMP shown to be released during alcohol-dependent pathogen-induced pulmonary inflammation (36). To evaluate the role of HMGB-1 and other proinflammatory cytokines in our demonstration of increased inflammation and pathology, we measured the protein levels in BALF samples from each treatment group via ELISA (**Figure 4**). As hypothesized, animals treated with co-exposure to EtOH and CB-agonist + *K. pneumoniae* had the greatest amount (in pg/mL) of all the proinflammatory cytokines that we measured. Most interestingly, this response is diminished in animals treated with both CB1R and CB2R antagonists, suggesting a role in CBRs in pathogen-induced pulmonary inflammation. These results suggest that CB1R and CB2R play a key regulatory role in the activation of the proinflammatory genes and stand at the crossroad of the EtOH-induced signaling pathways that lead to the activation of the proinflammatory genes.

**Figure 5:**
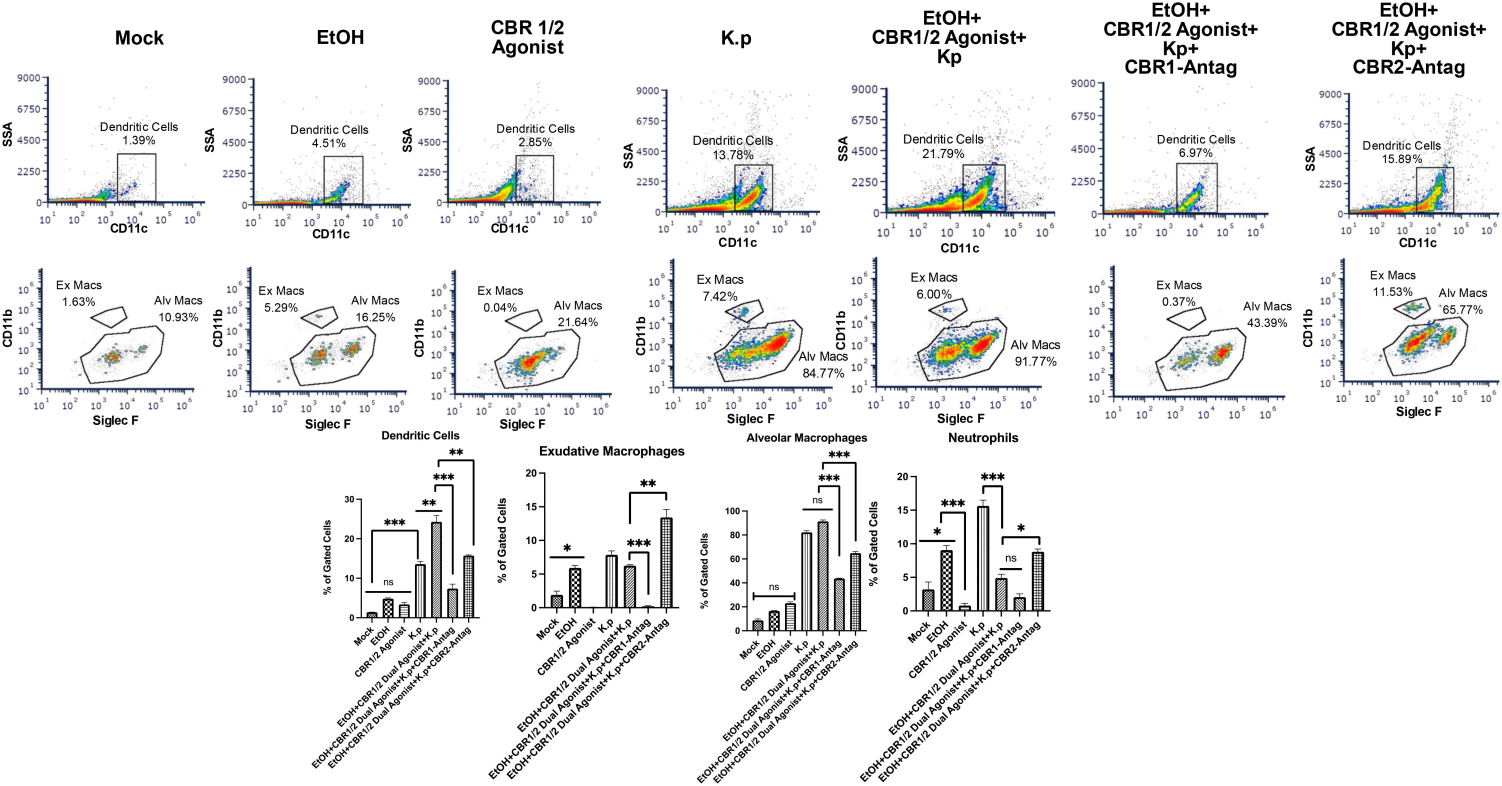
Increased immune cells in BALF from EtOH+ *Synthetic cannabinoid receptor agonist*-exposed mice measured by flow cytometry. BALF cells were isolated and stained with fluorophore-conjugated antibodies to identify key pulmonary immune cell subsets, including dendritic cells, exudative macrophages, alveolar macrophages, and neutrophils. The following antibodies were used: CD170 (Siglec-F)-FITC, Ly6G-PE, F4/80-AF700, CD11b-APC, CD11c-PC5.5, and a fixable viability dye (eFluor 450) (all from Thermo Fisher Scientific/eBioscience). Samples were acquired on a three-laser CytoFLEX flow cytometer (Beckman Coulter) and analyzed using FCS Express (version 7; DeNovo Software). At least 30,000 events were collected per sample. Gating strategies were applied to identify specific innate immune cell populations involved in pulmonary inflammation across treatment groups. Flow analyses per group, with a representative image for each group, are shown. *=0.05, **=0.001 and ***=0.0001. ns, not significant.

Lastly, following the treatment regime outlined in **Figure 1B**, we performed flow cytometry to evaluate BALF immune cells. The gating strategy is outlined in **Supplementary Figure S1**. Similar to our cytokine data, animals treated with co-exposure to EtOH and CB-agonist + *K. pneumoniae* had the greatest number of dendritic cells, macrophages, and neutrophils. Most interestingly, this response is diminished in animals treated with both CB1R and CB2R antagonists (**Figure 5**, **Supplementary Figure S2**).

## Discussion

The goals of the present study were to evaluate the inflammatory cytokine/chemokine profile from adolescent mice treated with synthetic cannabinoid receptor agonist (WIN55212-2) using a microbial challenge mouse model, to evaluate lung pathology from adolescent mice treated with synthetic cannabinoid receptor agonist (WIN55212-2), to evaluate the effects of cannabinoid exposure on the pulmonary inflammatory response, and to evaluate the effects of EtOH and cannabinoid co-exposure on the pulmonary inflammatory response. We show that CB pre-exposure and EtOH + CB co-exposure exacerbate *K. pneumoniae*-induced lung pathology. Lungs from animals treated with *K. pneumoniae*, synthetic cannabinoid receptor agonist (WIN55212-2) agonist, and cannabinoid receptor antagonists showed a diminishment in protein expression for IL-1β, IL-6, and IL-17, suggesting a role for CBRs in pathogen-induced inflammation that can be reversed when the receptors are blocked.

G-CSF is a glycoprotein that stimulates the bone marrow to produce granulocytes and release them into the bloodstream. It has also been hypothesized that G-CSF mediates brain repair by interacting with the endocannabinoid system (43). GM-CSF is a glycoprotein secreted by macrophages, T cells, and natural killer cells that serves as a proinflammatory cytokine. Unlike G-CSF, which exclusively promotes neutrophil proliferation, GM-CSF affects more cell types, predominantly, macrophages (44). GM-CSF has also been shown to increase the CB2R mRNA levels in microglia (45). Interestingly, the levels of G-CSF (in pg/mL) were lowest in animals treated with *K. pneumoniae* + CB2R-antagonists. MCP-1 regulates the migration and infiltration of macrophages (46). In addition, stimulation of CB2R has been shown to induce MCP-1 gene expression (47). MIP-1α and MIP-1β are secreted by macrophages and help to recruit other inflammatory cells during an immune response (48, 49). Although cannabinoids have, in some cases, been shown to decrease the levels of proinflammatory cytokines, MCP-1 and TNF-α have been shown to increase in response to cannabinoids (50). Fitting with our hypothesis, the levels of MCP-1, MIP-1α, and MIP-1β (in pg/mL) were greatest in mice treated with CB-agonist + *K. pneumoniae* but statistically lower in mice treated with CB-agonist + *K. pneumoniae* + CBR-antagonists.

Cannabinoid receptors CB1 and CB2 are G protein-coupled receptors with distinct yet overlapping roles in immune modulation. CB1 is primarily expressed in the central nervous system and is also present on peripheral immune cells. Its activation has been shown to promote pro-inflammatory signaling through the degradation of IκB, resulting in NF-κB nuclear translocation and transcription of inflammatory genes. In contrast, CB2 is predominantly expressed on immune cells and is generally associated with anti-inflammatory effects, including the suppression of TLR signaling and inhibition of NLRP3 inflammasome activation. However, CB2 can also support immune cell recruitment during early infection, reflecting its context-dependent role in inflammation.

The differential roles of CB1 and CB2 receptors in immune regulation are increasingly recognized as context-dependent and pathway-specific. Activation of CB1 has been shown to promote pro-inflammatory responses through the degradation of IκB, which facilitates NF-κB nuclear translocation and transcription of cytokines such as IL-6, TNF-α, and IL-1β. CB2 activation is generally associated with anti-inflammatory signaling, including the suppression of Toll-like receptor (TLR) pathways and inhibition of the NLRP3 inflammasome, reducing the levels of cytokines like IL-1β and DAMPs such as HMGB-1. In our study, the pharmacologic blockade of both CB1R and CB2R led to a reduction in inflammatory cytokines and DAMP expression, suggesting that under conditions of combined alcohol and synthetic cannabinoid exposure, both receptors may contribute to heightened pulmonary inflammation. One potential explanation is that chronic activation of these receptors—particularly CB1R—may dysregulate homeostatic immune signaling, shifting their roles from modulatory to pro-inflammatory in this pathological context. These findings highlight the importance of receptor signaling balance and raise the possibility that cannabinoid receptor antagonism may have therapeutic potential in substance co-exposure-driven lung inflammation.

The most evident evidence of a role for synthetic cannabinoid receptor agonist (WIN55212-2) receptors within pulmonary inflammation was observed from the histopathology of the lungs and modulation of immune cell profiles. We observed neutrophil chemotaxis in the lungs of *K. pneumoniae*-infected animals, inflammation surrounded by multi-nucleated cells, and alveoli thickening. Interestingly, the inflammatory response was increased in synthetic cannabinoid receptor agonist (WIN55212-2)-exposed animals challenged with *K. pneumoniae*. Even more compelling was that the neutrophilic influx was almost completely abrogated in the lungs of animals pre-treated with CBR antagonists. In addition, we observed that immune cells are increased in animals exposed to co-exposed to EtOH and CBs and challenged with a microbial pathogen. However, this response is mitigated with the use of CBR antagonists, suggesting therapeutic potential. The CBR1 is sometimes associated with pro-inflammatory pathways. Our results show that when CBR1 is blocked, it reduces the production of pro-inflammatory cytokines like TNF-α, IL-1β, and IL-6, which are key players in lung inflammation. While CBR2 is generally considered anti-inflammatory, its activation can sometimes increase immune cell recruitment to inflammation sites. Our results suggest that CBR2 may limit immune cell infiltration (macrophages, neutrophils) into lung tissue, reducing localized inflammation. Cannabinoid receptor activation, especially CBR1, can also stimulate the NF-κB pathway, which drives the transcription of pro-inflammatory genes. Blocking these receptors reduces NF-κB activation, thereby lowering the production of inflammatory mediators. In addition, cannabinoid receptors can crosstalk with other pathways, such as those mediated by TLRs and the inflammasome. Our results suggest that blocking cannabinoid receptors can dampen the amplification of inflammation-mediated through these pathways. It is also important to mention that blocking CBRs may directly modulate the activity of lung-resident immune cells, such as alveolar macrophages and dendritic cells, by reducing their pro-inflammatory cytokine production and antigen-presenting activities. These mechanisms underline the complex role of CBRs in inflammation and their potential as therapeutic targets for lung diseases. While CB2 receptor signaling is broadly regarded as anti-inflammatory, its role in immune regulation is highly context-dependent. In addition to suppressing pro-inflammatory cytokine production and inflammasome activation, CB2 can also facilitate immune cell recruitment, particularly during the early stages of infection. This dual functionality likely reflects a temporally regulated response, where CB2 signaling initially promotes host defense through cell trafficking but later contributes to the resolution of inflammation. In our study, blockade of CB2R led to a modest but detectable reduction in chemokine levels, suggesting impaired immune cell recruitment. These findings support a nuanced role for CB2 in pulmonary immune responses, especially under conditions of cannabinoid and alcohol co-exposure.

Both plant-derived (e.g., from *Cannabis sativa*) and endogenous (such as anandamide [AEA] and 2-arachidonoylglycerol [2-AG]) types have demonstrated immunomodulatory and anti-inflammatory roles in various disease models, including acute lung injury and asthma in C57BL/6 mice. COVID-19, caused by SARS-CoV-2, can lead to acute respiratory distress syndrome (ARDS) due to an overactive immune system. Cytokine storm is a crucial factor in ARDS, and current treatments are ineffective. Targeting cannabinoid receptors and endocannabinoids may offer a novel approach. SARS-CoV-2 binds to ACE2 receptors in various tissues, triggering immune responses that can clear the virus. However, in severe cases, this immune response becomes dysregulated, leading to ARDS and cytokine storm. Studies have shown that COVID-19 patients with severe infections exhibit elevated levels of inflammatory cytokines. Cannabinoids, such as THC and CBD, possess anti-inflammatory properties by acting through multiple pathways and may suppress the cytokine storm. Animal studies have demonstrated that THC can effectively prevent cytokine storms and ARDS, suggesting a potential therapeutic option. Targeting CB2 receptors or endocannabinoids could provide therapeutic benefits. While clinical trials with cannabinoids are limited due to their classification, they hold promise for treating ARDS. Developing new anti-inflammatory therapies is crucial in combating COVID-19 mortality, and cannabinoids could be a valuable tool in this fight (51). In addition, a study by Vuolo et al. delved into the effects of cannabidiol (CBD) treatment on allergic asthma, investigating its impact on airway inflammation, fibrosis, and cytokine levels. In the study, CBD reduced the airway hyperresponsiveness in Balb/c mice with allergic asthma induced by ovalbumin exposure. The results indicated that CBD treatment decreased the collagen fiber content in the airways and alveolar septa, along with markers associated with inflammation. The study also evaluated the expression of CB1 and CB2 receptors in asthmatic individuals, highlighting a significant inverse correlation between CB1 levels and lung function (52).

HMGB-1 (high mobility group box 1) is a known damage-associated molecular pattern (DAMP) that is released during tissue injury and inflammation, including alcohol-induced epithelial and immune stress. HMGB1 can amplify inflammatory responses and promote immune cell recruitment, which aligns with the cytokine changes and macrophage phenotypes that we observed. NF-κB is a central transcriptional regulator of pro-inflammatory genes. NF-κB signaling can be activated by both pathogen-associated stimuli (*K. pneumoniae*, for example) and endogenous factors such as HMGB1 and is known to be modulated by cannabinoid receptor activation. We propose that altered NF-κB activation may underlie the dampened or exaggerated cytokine responses seen in co-exposed mice.

It is crucial to acknowledge the limitations of our study. We primarily used a synthetic cannabinoid receptor agonist (WIN55212-2) in our experiments. In real-world scenarios, humans consume cannabis in whole forms, such as smoking, vaping, or edibles. The effects of isolated cannabinoid agonists may not fully encapsulate how cannabis impacts the immune system, lungs, or brain when consumed in its natural form. Although THC would have been ideal, we used WIN55212–2 because it is commercially available and used by many other labs for cannabinoid research. WIN55212–2 is not approved for clinical use in humans but is widely used in preclinical and basic research to study the endocannabinoid system and explore CB1/CB2-mediated signaling. Unlike natural cannabinoids like THC or CBD, WIN55212–2 is a synthetic compound with a distinct chemical structure, and due to its potency and receptor selectivity, it is a useful pharmacological tool to dissect CB1 vs. CB2 signaling *in vitro* and *in vivo* as demonstrated by our lab and others. The CB-agonists and antagonists that we used in our study were soluble in specific carriers (e.g., DMSO, ethanol, saline), making them easier to inject and administer orally in controlled doses. Whole cannabis requires combustion, vaporization, or extraction, leading to variability in administration and absorption. In addition, when using specific CB1/CB2 agonists, we were able to co-administer antagonists to confirm receptor-specific effects. There remain several gaps in research regarding cannabinoid receptors and their role in the inflammation of peripheral organs. However, our data with CBR antagonists demonstrates the ability to inhibit proinflammatory cytokine production in the reduction of IL-6, HMGB-1, TNF- α, and other cytokines. We also show that CBR antagonists reduced the effects of EtOH on proinflammatory cytokines after AIE exposure.

Another limitation of this study is the absence of clinical respiratory function measurements, such as airway resistance, conductance, and peak flow parameters. These metrics, typically assessed using whole-body plethysmography, would offer valuable functional insights into how co-exposure to WIN55212–2 and alcohol affects pulmonary physiology. We acknowledge the significance of correlating immune responses with changes in lung function and plan to incorporate these clinically relevant respiratory parameters in future studies as our capabilities expand. This future direction will enhance the translational relevance of our findings and provide a more comprehensive understanding of the impact of cannabinoid and alcohol co-exposure on lung health. Another limitation of our study is the absence of *in vitro* experiments using immune cells isolated from secondary lymphoid organs, such as the spleen or mediastinal lymph nodes. While our current work focused on *in vivo* lung immune responses, particularly the innate compartment during early bacterial infection, *ex vivo* stimulation of splenocytes or lymph node-derived cells with WIN55212–2 would provide valuable mechanistic insights into how this synthetic cannabinoid receptor agonist directly influences lymphocyte activation, cytokine production, and receptor-mediated signaling pathways. Future studies will incorporate these *in vitro* approaches to better define the systemic immunomodulatory effects of WIN55212–2 and further delineate its impact on adaptive immune cells beyond the lung environment.

A final limitation of our study is the lack of a detailed analysis of adaptive immune cell populations, such as CD4^+^ T cells, CD8^+^ T cells, and NKT cells. Our flow cytometry panels were intentionally designed to focus on antigen-presenting cells and other innate immune subsets, including alveolar and interstitial macrophages and dendritic cells to characterize the early innate immune response to co-exposure with alcohol, cannabinoid receptor agonist, and *K. pneumoniae*. In line with this goal, we used whole-lung enzymatic digestion instead of Histopaque gradient separation to preserve the native proportions of lung-resident innate cells. However, we acknowledge that isolating lung mononuclear cells using density gradient centrifugation and incorporating lymphoid panels would provide valuable insights into the adaptive immune response. Future studies will incorporate these approaches to comprehensively assess how co-exposure affects both innate and adaptive immune compartments within the lung.

In the current study, we showed that endocannabinoid (eCB)-CB1R axis-induced signaling also plays a key role in modulating EtOH-mediated inflammatory expression. Our findings of reversal of protein gene expression by CBR antagonists open up a novel therapeutic approach to target CBRs to mitigate peripheral detrimental immune activation within the lung. Several studies from different laboratories have suggested that CBRs play a role in different immune functions (53–58). Thus, these results suggest a novel interaction of EtOH with the CBR signaling and resulting cellular immune activation in the context of the lung.

Previously, our lab has shown that EtOH modulates the expression of cannabinoid receptors 1 and 2 on macrophages within the lung. We showed the novel finding that EtOH activates and primes lung macrophages to a more severe inflammatory response when challenged with a microbial pathogen, and this response is mediated through CBRs. We have shown here that CB1R antagonist AM6545 and CB2R antagonist SR144528 both demonstrated the ability to inhibit proinflammatory cytokine production after microbial challenge. Further research is needed to determine how EtOH and cannabinoids are interacting mechanistically with pathways in the lung, leading to the upregulation of proinflammatory cytokines after microbial challenge. The overarching goal of this study was to address critical barriers to progress in the scientific field by addressing three critical barriers: (1) lack of knowledge regarding the effects of polysubstance use of the two most commonly used comorbid drugs of abuse, namely, cannabinoid and EtOH during adolescence on pulmonary pathobiology; (2) identifying the effects of this co-exposure and defining the critical players involved in the response will provide new drug targets for pulmonary inflammation beyond the traditional approach; and (3) very little is known regarding alcohol and cannabis co-exposure.

Our primary objective in our study was to characterize the immune response in the context of concurrent exposure to synthetic cannabinoid receptor agonist (WIN55212-2) agonist and alcohol, followed by a challenge with *Klebsiella pneumoniae* infection. Our approach involved measuring cytokine profiles and immune cell phenotypes in response to these exposures to gain insights into how these factors impact pulmonary immune responses. Importantly, our findings provide crucial foundational data by identifying key changes in cytokine expression and immune cell populations in response to these exposures. This information serves as a basis for future mechanistic studies to explore the specific pathways involved, such as CB1/CB2 receptor signaling, alcohol-induced immune modulation, or bacterial immune evasion strategies. Based on existing literature and our findings, several potential mechanisms may explain how cannabinoid agonists and alcohol influence pulmonary immune responses during *Klebsiella pneumoniae* infection. Alcohol disrupts alveolar macrophage function, altering pathogen recognition and cytokine production (13). It also influences epithelial barrier integrity, increasing susceptibility to bacterial invasion and modifying immune cell recruitment. In addition, co-exposure to alcohol and synthetic cannabinoid receptor agonist (WIN55212-2) can lead to differential immune modulation, either exacerbating or dampening inflammatory responses, some of which we have shown in our study. We hypothesize that this could involve changes in NF-κB signaling, inflammasome activation, or epigenetic modifications in immune cells. Lastly, considering our interest in HMGB-1, it is plausible that exposure to alcohol and cannabinoids modulates HMGB-1 release and signaling via the RAGE receptor, potentially impacting the inflammatory response to *Klebsiella pneumoniae*. This pathway could either contribute to excessive inflammation or immune suppression, depending on the specific receptor activation profile. To further explore these potential mechanisms, future studies should include RT-PCR analyses to assess the activation of NF-κB, HMGB1, and inflammasome pathways in lung tissues and RNA sequencing or proteomic approaches to identify transcriptional and post-translational changes in immune cells following exposure. While our current study provides important foundational data on immune phenotypes and cytokine profiles, these future mechanistic investigations will help uncover the precise molecular pathways involved in alcohol- and cannabinoid-induced immune modulation during pulmonary infection.

## Data Availability

The raw data supporting the conclusions of this article will be made available by the authors, without undue reservation.

## Supplementary material

Supplementary Figure 1 Flow cytometry gating strategy. BALF cells were first gated on single, live cells using forward and side scatter properties and exclusion of the viability dye (eFluor 450). Immune cell subsets were then identified based on surface marker expression as follows: alveolar macrophages (CD11c^+^ Siglec-F^+^), exudative macrophages (CD11b^+^ F4/80^+^), dendritic cells (CD11c^+^), and neutrophils (CD11b^+^ Ly6G^+^). Representative plots are shown for each gating step. A minimum of 30,000 events was collected per sample, and the gates were set using single-colored controls.

Supplementary Figure 2 Identification of pulmonary immune cell subsets in BALF across experimental groups using flow cytometry. Representative gating plots are shown for each treatment group to illustrate the identification of key innate immune cell populations in bronchoalveolar lavage fluid (BALF), including dendritic cells, exudative macrophages, alveolar macrophages, and neutrophils. Gating was performed using single-colored controls to ensure accuracy. Representative plots are shown from the following experimental groups: mock, ethanol, CBR agonist (WIN55212-2), *K. pneumoniae*, ethanol + CBR agonist + *K. pneumoniae*, ethanol + CBR agonist + *K. pneumoniae* + CBR1 antagonist, and ethanol + CBR agonist + *K. pneumoniae* + CBR2 antagonist.
