# Supplementary figures and images for: Cannabinoids and alcohol co-exposure modulate pathogen-induced pulmonary immune responses
Source: Front Immunol. 2025 Jul 7;16:1539813. doi: 10.3389/fimmu.2025.1539813 (PMC12277145; doi:10.3389/fimmu.2025.1539813)

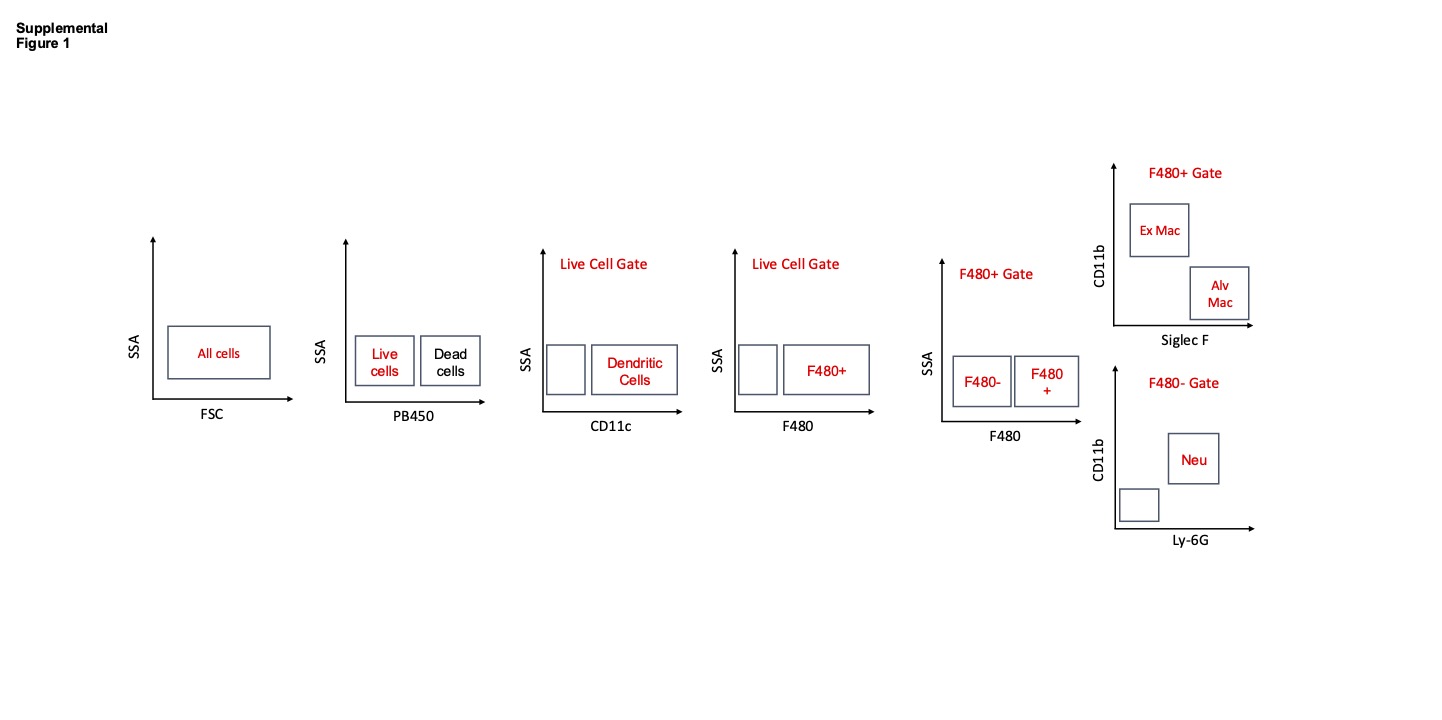

Supplement: Supplementary Figure 1 — Flow cytometry gating strategy. BALF cells were first gated on single, live cells using forward and side scatter properties and exclusion of the viability dye (eFluor 450). Immune cell subsets were then identified based on surface marker expression as follows: alveolar macrophages (CD11c+ Siglec-F+), exudative macrophages (CD11b+ F4/80+), dendritic cells (CD11c+), and neutrophils (CD11b+ Ly6G+). Representative plots are shown for each gating step. A minimum of 30,000 events was collected per sample, and the gates were set using single-colored controls. [file Image1.jpeg]

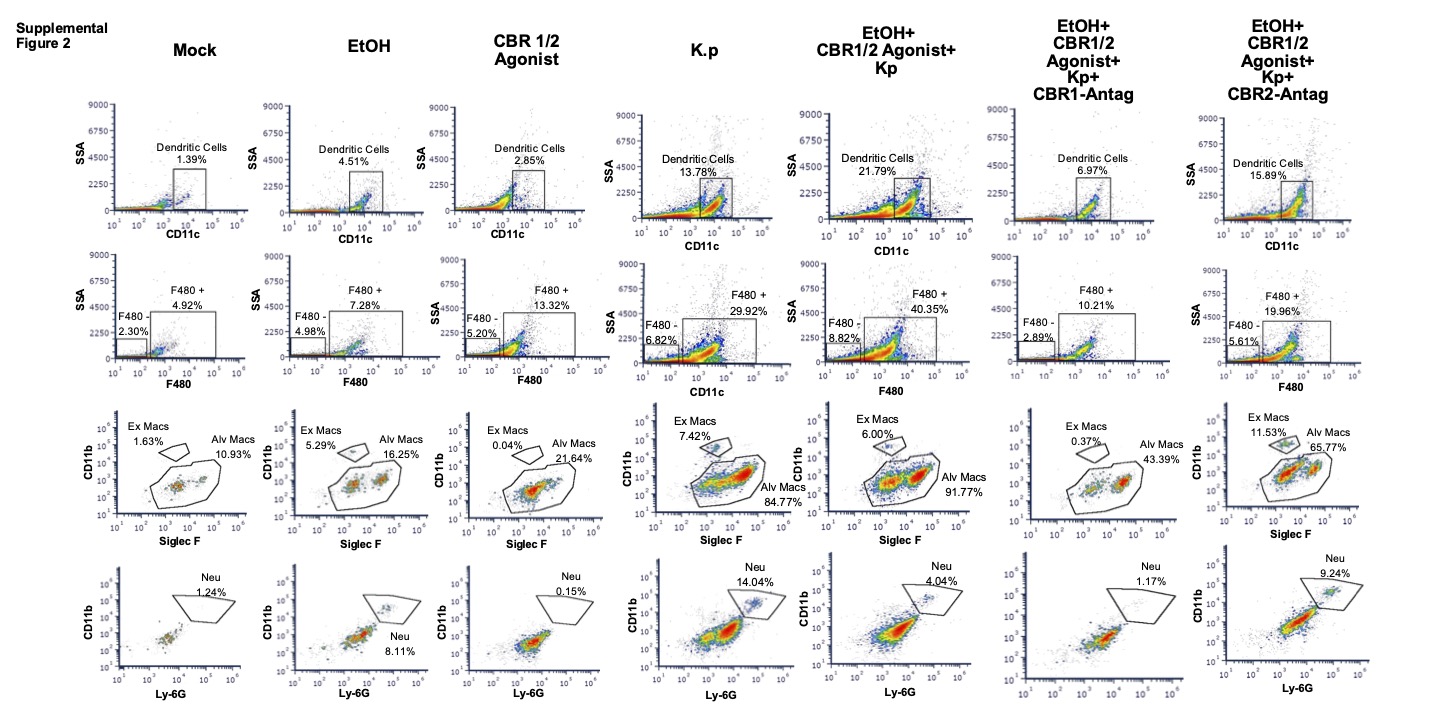

Supplement: Supplementary Figure 2 — Identification of pulmonary immune cell subsets in BALF across experimental groups using flow cytometry. Representative gating plots are shown for each treatment group to illustrate the identification of key innate immune cell populations in bronchoalveolar lavage fluid (BALF), including dendritic cells, exudative macrophages, alveolar macrophages, and neutrophils. Gating was performed using single-colored controls to ensure accuracy. Representative plots are shown from the following experimental groups: mock, ethanol, CBR agonist (WIN55212-2), K. pneumoniae, ethanol + CBR agonist + K. pneumoniae, ethanol + CBR agonist + K. pneumoniae + CBR1 antagonist, and ethanol + CBR agonist + K. pneumoniae + CBR2 antagonist. [file Image2.jpeg]
